# Editorial: Magnetic resonance-guided laser interstitial thermal therapy (MRg-LiTT) in the minimally invasive surgical treatment of epilepsy and/or brain neoplasms

**DOI:** 10.3389/fneur.2024.1378585

**Published:** 2024-02-12

**Authors:** Alessandro Consales, Jesse Skoch, Zulma Tovar-Spinoza

**Affiliations:** ^1^Division of Neurosurgery, IRCCS Istituto Giannina Gaslini, Genoa, Italy; ^2^Division of Pediatric Neurosurgery, Cincinnati Children's Hospital Medical Center, Cincinnati, OH, United States; ^3^Department of Neurosurgery, SUNY Upstate Medical University, Syracuse, NY, United States

**Keywords:** MR-gLiTT, epilepsy surgery, brain tumor surgery, stereoelectroencephalography (SEEG), minimally invasive neurosurgery

Magnetic Resonance-guided Laser interstitial Thermal Therapy (MR-gLiTT) has been a significant breakthrough in minimally invasive neurosurgery over the past decade. It has brought about a revolutionary change in how we treat epilepsy and has shown promising results in neuro-oncological surgery (1–3).

MR-gLiTT is considered highly effective in treating hypothalamic hamartoma, becoming one of the standard indications for this technology, which poses significant challenges to traditional neurosurgical methods (1, 4, 5). This technique also provides a therapeutic alternative, with demonstrated safety and efficacy, to established surgical approaches for other epileptogenic conditions.

MR-gLiTT offers several advantages over traditional techniques, including the ability to treat deep brain lesions with reduced surgical complication rates, a shorter postoperative hospital stay, it can be repeated or staged, lesser disruption of daily activities, faster recovery, and more pleasant aesthetic results (6, 7).

This Research Topic includes six papers that explore a range of topics, from treating deep brain lesions, which are challenging to address with more aggressive approaches, to testing physics and addressing new therapeutic hypotheses.

Vetkas et al. conducted a systematic review on using MR-gLiTT in the minimally invasive treatment of epileptogenic and oncologic insular lesions. Their study is fascinating because it is the first in the literature on the indications, outcomes, and complications of MR-gLiTT related to the insula. The authors identified 10 retrospective studies that described a total of 53 patients, the vast majority of whom (87%) had epilepsy. The results reported were positive for epilepsy cases but also appreciable from the neuro-oncological point of view, with low and mostly transient adverse event rates. It will be important in the coming years to evaluate the outcomes of MR-gLiTT for insular lesions through randomized and controlled trials.

Spacca et al. retrospectively analyzed data from six pediatric patients with deep-seated brain tumors treated with MR-gLiTT at their center, discussing the indications and preparation for LiTT, technical issues, clinical, and radiological follow-up, impact on quality of life, and oncological treatment. The results reported by the authors, albeit obtained on a small case series, are encouraging and stimulate further investigation into the possibility of treating selected cases of deep brain lesions minimally invasively with good results from an oncological point of view and a low incidence of complications.

Hypothalamic hamartoma (HH) is a rare intracranial disease whose manifestations include gelastic seizures and precocious puberty. The diagnosis and treatment of HH have changed substantially over the past three decades. Lu et al. carried out a bibliometric analysis over the past 30 years (1992–2021), revealing the evolution and development of this Research Topic. It is clear from the bibliometric research by Lu et al. that the treatment trend for this rare condition is increasingly oriented toward minimally invasive techniques, among which MR-gLiTT will play a prominent role.

The results of MR-gLiTT depend on the laser wavelength, duration, and power delivered. Laser wavelengths of 980 nm and 1,064 nm applied to surgical procedures have been described in the literature. The 1,064 nm wavelength laser is a more recent introduction. It is widely used to treat epilepsy and brain tumors owing to its advantages in deeper penetration and the larger ablation zone. Since there is no numerical model that simulates its thermal profile and ablation effect in brain tissue with accurate *in vivo* validation, Cao et al. developed and validated such a model, demonstrating its feasibility and reliability in predicting the ablation area, which is essential for presurgical planning.

Another issue covered in this Research Topic concerns the minimally invasive treatment of focal epilepsies associated with focal cortical dysplasias. Li et al. conducted a meta-analysis to evaluate and compare the efficacy and safety of MR-gLiTT and Stereo-Electro-Encephalo-Graphy (SEEG)-guided Radio-Frequency Thermal-Coagulation (SEEG-RFTC) in patients with this type of epilepsy. The authors found no statistically significant difference between the two treatment methods, with good seizure outcome and low incidence of complications for both. However, MR-gLiTT, a more recently adopted technique in neurosurgery, still has room for growth and the undoubted advantage of real-time control of the lesions produced. Finally, Guida et al. described two patients with NF1 and Moyamoya angiopathy who had already undergone cerebral indirect revascularization and subsequently underwent MR-gLiTT for low-grade brain neoplasms in order to avoid compromising the outcome of cerebral revascularization possibly caused by craniotomy. This report of two cases demonstrates how MR-gLiTT can be an effective alternative to open surgery in cases where traditional surgery may be burdened with significant complications.

In conclusion, MR-gLiTT is a viable option for various epileptogenic conditions, complementing established surgical techniques (7). This Research Topic provides valuable insights into the versatility and effectiveness of MR-gLiTT, and we hope it will be of great interest to our readers.

## Author contributions

AC: Conceptualization, Methodology, Supervision, Writing—original draft, Writing—review & editing. JS: Writing—original draft, Writing—review & editing. ZT-S: Writing—original draft, Writing—review & editing, Supervision.
